# Genetic Testing of Children for Predisposition to Mood Disorders: Anticipating the Clinical Issues

**DOI:** 10.1007/s10897-014-9710-y

**Published:** 2014-08-01

**Authors:** Jessica A. Erickson, Lili Kuzmich, Kelly E. Ormond, Erynn Gordon, Michael F. Christman, Mildred K. Cho, Douglas F. Levinson

**Affiliations:** 1 Center for the Integration of Research on Genetics and Ethics of the Stanford Center for Biomedical Ethics, Stanford University, Stanford, CA USA; 2 Center for Reproductive Health, University of California San Francisco Medical Center, San Francisco, CA USA; 3 Department of Genetics, Stanford University, Stanford, CA USA; 4 Coriell Personalized Medicine Collaborative, Coriell Institute for Medical Research, Camden, NJ USA; 5 Department of Pediatrics, Stanford University, Stanford, CA USA; 6 Department of Psychiatry and Behavioral Sciences, Stanford University, 401 Quarry Rd., Rm. 3322, Stanford, CA 94305 USA

**Keywords:** Genetic testing, Children, Benefits, Risks, Positive predictive value, Mood disorders, Health Belief Model, Mood disorders

## Abstract

Large-scale sequencing information may provide a basis for genetic tests for predisposition to common disorders. In this study, participants in the Coriell Personalized Medicine Collaborative (*N* = 53) with a personal and/or family history of Major Depressive Disorder or Bipolar Disorder were interviewed based on the Health Belief Model around hypothetical intention to test one’s children for probability of developing a mood disorder. Most participants (87 %) were interested in a hypothetical test for children that had high (“90 %”) positive predictive value, while 51 % of participants remained interested in a modestly predictive test (“20 %”). Interest was driven by beliefs about effects of test results on parenting behaviors and on discrimination. Most participants favored testing before adolescence (64 %), and were reluctant to share results with asymptomatic children before adulthood. Participants anticipated both positive and negative effects of testing on parental treatment and on children’s self-esteem. Further investigation will determine whether these findings will generalize to other complex disorders for which early intervention is possible but not clearly demonstrated to improve outcomes. More information is also needed about the effects of childhood genetic testing and sharing of results on parent–child relationships, and about the role of the child in the decision-making process.

**Electronic supplementary material:**

The online version of this article (doi:10.1007/s10897-014-9710-y) contains supplementary material, which is available to authorized users.

## Introduction

The primary motivation for molecular genetic research on bipolar disorder (BPD) and major depressive disorder (MDD) is to determine how genetic variation influences predisposition to illness, thus providing targets for improved treatment and perhaps prevention. Another outcome could be the development of genetic tests for diagnosis and for prediction of future illness. Mood disorders are only partially heritable (60–80 % for BPD, 35–40 % for MDD based on twin studies [Merikangas et al. 2002]), and it is unclear whether highly predictive tests can be developed, but it appears likely that large-scale sequencing-based information will permit the development of some form of risk profiling for common complex disorders. Regardless of the predictive power of the tests, in this information age it is likely that some consumers will want to obtain whatever information genetic tests can provide. Previous studies demonstrate that this interest extends to testing to identify children who are at increased probability of developing mood disorders (Jones et al. 2002; Laegsgaard et al. 2009; Meiser et al. 2005, 2008; Smith et al. 1996; Trippitelli et al. 1998).

Health professionals and ethicists are justifiably concerned about the commercialization of modestly predictive or invalid genetic tests for mood disorders (Braff and Freedman 2008), and that testing of children could alter parental behavior and the child’s self concept (Hoge and Appelbaum 2012) while producing harms including feelings of genetic “doom” and self-imposed limitations (Malpas 2008), reduced self-esteem (Borry et al. 2006), lack of respect for children’s autonomy and confidentiality (Fenwick 2010; Parker 2010; Corcoran et al. 2005), and employment or insurance discrimination. But some authors have noted potential benefits: early detection, tailored medication and reduced stigmatization by self and by family members (Erickson and Cho 2011; Hoop et al. 2010; Laegsgaard et al. 2009; Meiser et al. 2008; Miklowitz and Chang 2008). Professional society guidelines advocate avoiding genetic testing of children for adult-onset diseases unless early and effective treatment is available (American Society of Human Genetics 1995; Borry et al. 2006; Parker 2010; Ross et al. 2013; National Society of Genetic Counselors 2012). However, mood disorders often start during childhood or adolescence, and although treatment outcomes are highly variable, there are reports that early interventions using individual, family- or school-based therapy might improve the course of illness in children with early symptoms (Garber et al. 2009; Miklowitz and Chang 2008). These findings support the view of some parents that they could provide a more protective environment for at-risk children. Decisions about predictive genetic testing are expected to be complex because of the variability of perceptions about the potential benefits and risks of testing and about the disorder itself (e.g., due to the wide range of ages at onset, severity and degree of recurrence or chronicity) (Meiser et al. 2005). It has been suggested that parents and healthcare providers might best make genetic testing decisions about the testing of minors on a case-by-case basis (Fenwick 2010). This view is reflected in the recent American College of Medical Genetics and Genomics report on genetic testing of children (Ross et al. 2013), which suggests that the negative psychosocial effects of genetic test information on children and adolescents may have been overstated in older literature (Wade et al. 2010), and that decisions about predispositional genetic testing (for disorders with decreased penetrance and without definitive treatment) may be based on multiple factors that determine the child’s “best interest.”

Therefore, whether or not genetic testing for predisposition to mood disorders in children will have clear benefits in the foreseeable future, we assume that it will occur, and probably initially in a precipitous fashion -- indeed, until recently, the personal genomics company 23andMe did provide information about genotypes that are weakly associated with risk of BPD (https://doi.org/www.23andme.com) although the effect size of these variants is very small. The present study has attempted to take an additional step toward understanding attitudes toward future genetic tests that might assess children’s probability of developing a mood disorder. Previous studies have probed adults’ attitudes toward genetic tests that could determine the future probability of illness with a high or unspecified degree of certainty (Laegsgaard et al. 2009; Smith et al. 1996) Here, we also ask adult participants to consider whether they would use tests with a modest level of predictive power, while inquiring about a broader range of attitudes regarding when such tests should be made accessible and potential pros and cons of testing. Our goal has been to use the theoretical framework of the Health Belief Model (HBM) (Janz et al. 2002) to explore more deeply the issues and risks that will arise for parents and their children who wish to obtain genetic information for risk of mood disorders, so as to prepare and better equip clinicians to deal with these issues . The theory-based findings from this study might contribute to the design and implementation of effective and appropriate communication strategies about genetic testing for risk of mood disorders in children.

An inherent difficulty in research on intentions is the need to use hypothetical scenarios. Previous research shows that fewer people actually use genetic tests than had been predicted by studies of attitudes towards hypothetical tests (Lerman et al. 2002). We attempted to narrow the gap between hypothetical and actual behavior (Ajzen et al. 2004) by studying individuals who had personal and/or family histories of mood disorders and who were already participating in a personalized medicine research program which included genetic testing for common diseases (e.g., heart disease, diabetes and melanoma). Previous studies have demonstrated a strong correlation between interest to test and family history of the disorder (Wilde et al. 2011; Lerman et al. 1994). Together, both of these personal attributes of our participants might make their attitudes more predictive of whether individuals who are “early adopters” of personalized medicine in the future would pursue genetic testing of children for mood disorders. Their attitudes might therefore anticipate the issues that genetic counselors could confront when such tests become available.

Note that we use the terms “probability,” “predisposition” and “susceptibility” rather than “genetic risk,” except in the Discussion when referring to the standard medical and research use of the latter term. Perceptions of “risk” reflect an individual’s ideas about the numerical probability as well as the severity of the outcome (Austin et al. 2012). Undergoing genetic tests and learning the results are also considered to have potential “risks” in the sense of adverse effects. To avoid confusing these meanings, we have attempted to keep them separate here.

## Materials and Methods

### Institutional Approval

The protocol was approved by the Institutional Review Boards of Stanford University and the Coriell Institute for Medical Research.

### Recruitment

Study participants who were 18 years or older were recruited from among the participants in the Coriell Personalized Medicine Collaborative (CPMC), a prospective observational study of whether personalized genetic information can be used to improve health. An email was sent to a random sample of 250 CPMC participants (10 % of the active CPMC population as of 12/21/2010; unselected with regard to medical or psychiatric history), inviting any participants with a personal and/or family history (first- or second-degree relative) of MDD or BPD to be interviewed for a Stanford research study examining attitudes toward genetic testing for mood disorders. Interested individuals followed a link to a more detailed online announcement (within the password-protected CPMC member space). Those who affirmed a personal and/or family history were asked to electronically sign the informed consent document to proceed with the study. Stanford-based interviewers attempted to reach the 82 individuals who consented online, of whom 53 (65 %) were successfully contacted, verbally confirmed their informed consent and completed the interview.

### Measure

**Fig. 1:**
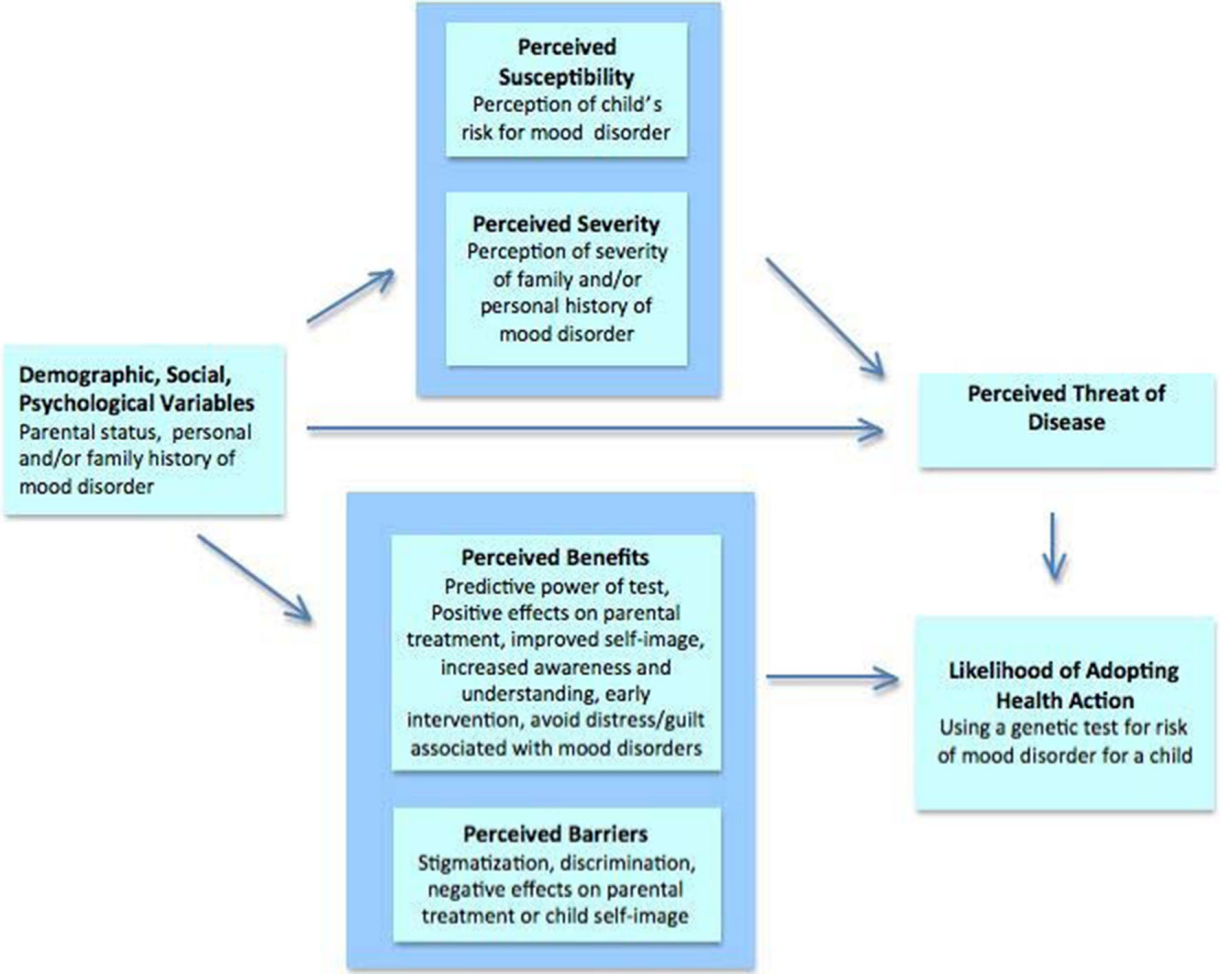
Title: Applying the Health Belief Model to interest in pediatric genetic testing for risk of developing mood disorders

We developed a semi-structured interview schedule for this study. We first developed a framework, based on the HBM (Janz et al. 2002), for predicting behavior based on perceived susceptibility (for oneself or for one’s child), severity (of the disorder) and benefits and barriers/risks of testing (Fig. 1). We drew upon a previous questionnaire (Meiser et al. 2008) to develop an initial list of perceived potential benefits and risks of genetic testing for mood disorders, and then wrote our own set of items (the schedule is provided as a supplementary file) to collect information about demographics, self-reported personal and family history of mood disorders, beliefs about etiology, and attitudes towards future genetic testing for the probability of future mood disorders for oneself and for children. We focus here on results related to children. We obtained quantitative information by asking for a summary response to most items from a list of anchor points, *and* qualitative information (by encouraging comments about quantitative items and by including open-ended questions). We inquired separately about attitudes toward testing oneself and children with a highly vs. modestly predictive test.

For example, the interviewer introduced the section on perceived benefits and risks by saying, in part, “I would like to ask your opinion about predictions that some people have made about how genetic tests for mood disorders might be used in the future, and what their positive and negative effects might be.” The interviewer read each statement and asked a standard set of questions about each one; asked for a summary response based on the anchor points; and invited comments. For example, the interviewer read, “Genetic tests for mood disorders might permit earlier diagnosis,” and then asked, “How likely do you think this is?” (very unlikely, unlikely, neutral/unsure, likely, very likely—which were later re-coded as unlikely, unsure or likely); “if it turned out to be true, would this be a good or a bad thing” (very good, somewhat good, neutral/unsure, bad, very bad—re-coded as good, neutral, bad); and “if it turned out to be true, how do you think it would influence whether people like you would request such a test for yourself, and for a child?” (for self and child separately: negatively, no influence/unsure, positively). Later in the interview, participants were asked how likely they would be to test themselves or their child using a highly or modestly predictive test (a four-point scale was re-coded to interested vs. not interested), followed by open-ended questions about how it might help the person to know the results and what problems it would cause.

In initial interviews, we tried alternative ways to ask participants about how their level of interest in testing would be influenced by the certainty with which the test could predict future illness. We arrived at the following wording to introduce the concept of high vs. modest predictive power for a test: “Let’s imagine that some future genetic testing program could either predict with a high degree of certainty (let’s say 90 %) that someone would go on to develop a mood disorder, or could only predict with a moderate degree of certainty (let’s say 20 %) that someone would go on to develop a mood disorder.” Subsequently, we asked all questions about degree of interest in testing (for self or for children) separately for hypothetical tests with “high” or “moderate” certainty, using this definition. We recognize that “20 % certainty” is actually consistent with the predictive power of family history alone (thus we refer to this level here as “modest,” whereas we used “moderate” in most places in the interview), but we found that most participants did not seem to interpret these questions in a strictly quantitative way, and that this wording conveyed the difference between a test giving a very strong prediction vs. giving a rather small amount of information about the probability that *a given individual person* will become ill.

Interviews were carried out in equal numbers (26 and 27) by two trained interviewers. Interviews were then recorded and professionally transcribed. Audio and transcribed versions were crosschecked for accuracy. The range of qualitative responses was similar in the first and second halves of the sample, suggesting that we were capturing the most prevalent attitudes in this population.

### Data Analysis

A total of 103 coding categories were defined, corresponding to individual interview questions and themes of interest (see the supplemental file for details). Quotes that exemplify general themes are provided below. Note that qualitative responses have been included throughout the Results section to add depth to the quantitative results, but no formal qualitative analysis procedure was applied. There were no significant differences between the two interviewers in the distributions of quantitative ratings for individual items, therefore all interviews were analyzed as one group.

**Fig. 2:**
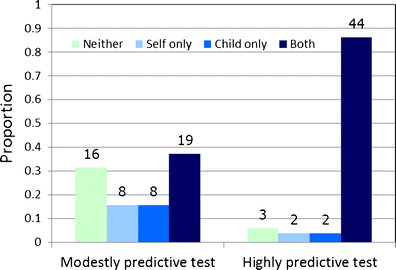
Interest in testing self and child: effect of predictive power of the test. **Legend**: Shown are the proportions of participants who expressed interest in genetic testing for mood disorders for self and/or a child, separately for a modestly predictive (“20 % certainty”) test (*N* = 52) and for a highly predictive (“90 % certainty”) test (*N* = 51 participants who answered both questions). Absolute counts are shown above each bar. The effect of predictive power was highly significant, e.g., for interest in testing children, *p* = 0.000021 (sign test). The proportion of participants who would test children (child only or self and child) was 53 % for a modestly predictive test (27/51) and 90 % for a highly predictive test (46/51)

Relationships between pairs of categorical variables were analyzed by Fisher’s exact tests (for 2 × 2 tables) or chi-square tests, focusing on possible predictors of interest in tests with modest predictive power since realistically future genetic tests for risk of mood disorders will likely be only minimally predictive. The significance of the difference between interest in highly and modestly predictive tests (Fig. 2) was analyzed with a sign test, for 51 subjects who responded to both questions.

## Results

### Demographics

Fifty-three interviews were completed and analyzed, i.e., 21 % of the invited population which was unselected for mood disorder history, and 65 % of the participants who consented to participate online. Participants were primarily Caucasian, female, over 51 years old and well educated (Table 1). Most (58 %) had a personal history, and all but five had a family history, of MDD or BPD. Most (66 %) perceived themselves or their family as severely affected by a mood disorder, although perceived severity did not predict interest in testing of children.

**Table 1:** Demographics and personal/family history of mood disorders

	Count	Percent
	(*N* = 53)	
Demographics		
Female gender	40	75 %
Self-reported race: Caucasian	50	94 %
Age 51 and over	33	62 %
Married	31	58 %
Have children	30	57 %
Education level		
Some or all of high school	3	6 %
Some or all of college	18	34 %
Some or all of graduate school	32	60 %
Personal and family history		
Personal history of Major Depressive Disorder	26	49 %
Personal history of Bipolar disorder I or II	5	9 %
Family history of mood disorder	48	91 %
Family history of suicide	12	23 %
Chronic mood disorder	15	28 %
Perceived impairment during worst mood episodes		
Moderate	9	17 %
Substantial	24	45 %
Perceived severity of family history of mood disorders		
Mild	19	36 %
Moderate	16	30 %
Severe	15	28 %
Perceived effect on self of family history of mood disorders		
Mild/No effect	19	36 %
Moderate	16	30 %
Severe	15	28 %

### Interest in Testing for Mood Disorder Risk for Self and/or Child

Most participants (89 %), regardless of whether they had a personal history of mood disorder,, were interested in having a genetic test themselves if the test could predict the future probability of mood disorder with 90 % certainty, while 53 % remained interested if only 20 % certainty could be achieved. (Although participants with mood disorders already knew their own diagnoses, this population was interested in testing of oneself to understand how genes might contribute to their illness.) Of those participants who were interested in highly predictive genetic tests for themselves, 85 % were also interested in testing their children. For modestly predictive tests, of the 51 participants who responded to both questions, most were interested in testing either themselves and their children (37 %, 19/51), or neither (31 %, 16/51) (Fig. 2). A slight majority of all participants stated that they felt free to act on their intentions to test self (57 %) and their children (53 %) regardless of others’ (spouses, family members, physicians) opinions. More than half (64 %) believed that other people would use genetic test results to decide whether to have children, with the entire sample roughly split as to whether this would be a “good” or “bad” use of testing.

### Personal Experience with Children and Mood Disorders

Of the 30 study participants with children, 20 (67 %) voiced concern that one or more of their children had a mood disorder or early symptoms (Table 2). Most participants (62 % of the total sample) perceived that their real or hypothetical offspring had an increased probability of developing a mood disorder compared to the average population. Seven participants (13 %) reported that they had decided not to have children for fear of transmitting genes that would increase their offspring’s probability of developing a mood disorder.

**Table 2:** Attitudes towards genetic testing of children for predisposition to mood disorders

	Count	Percent
	(*N* = 53)	
Concern about children demonstrating mood disorder symptoms	20	38 %
Perceived probability of children developing mood disorders:		
Same/less than the population average	14	26 %
More than the population average	33	62 %
Believed that future use of prenatal genetic testing is likely	28	53 %
Evaluation of using a prenatal genetic test to make termination decisions:		
Negative	33	62 %
Neutral/Unsure	14	26 %
Positive	6	11 %
**Total responses:**	**53**	
Believed that people are likely to use future genetic tests of mood disorder predisposition (in parent) to make childbearing decisions	34	64 %
Evaluation of use of genetic tests for mood disorders to make childbearing decisions:		
Negative	18	34 %
Neutral/Unsure	15	28 %
Positive	20	38 %
**Total responses:**	**53**	
Effect of genetic testing on how parents treated children is likely to be:		
Negative	13	25 %
Neutral/Unsure	19	36 %
Positive	21	40 %
**Total responses:**	**53**	
Effect of genetic testing on how child viewed self would be:		
Negative	28	53 %
Neutral/Unsure	20	38 %
Positive	5	9 %
**Total responses:**	**53**	
Best age to have a child tested		
At birth	11	21 %
Before the age of 12	34	65 %
During teens	2	4 %
Over 18	2	4 %
Only if there are symptoms/Never	3	6 %
**Total responses:**	**52**	
Best age to discuss genetic test results with a child (parents’ and non-parents’ responses):		
Depends on child/Unsure would share results	8	17 %
Only if symptoms were observed	8	17 %
At age of testing	5	10 %
Before or during teens	15	31 %
Over 18	12	25 %
**Total responses:**	**48**	

### Attitudes Towards Genetic Testing for Risk of Mood Disorders in Children

#### Predictive Power of a Test (High vs. Modest) Influences Interest in Testing

Participants were more likely to express interest in testing their children for mood disorders with a highly predictive test (87 %) compared with a modestly predictive test (51 %). The effect of the predictive power of the test was highly significant (sign test, *p* = 0.000021; see Fig. 2 for the raw data for this test). Two primary reasons were given for interest in testing: 1) increased awareness of possible symptoms and 2) the potential for early intervention. Participants were almost evenly divided between these two reasons for testing.

*“I think that [testing is] beneficial because if you know that [the child] has a high risk and… if they start exhibiting certain symptoms… you’re at least more aware… of certain signs and you’re able to get them help more quickly.”*

#### Positive and Negative Influences of Testing on Parental Treatment of Children

Participants mentioned that testing could either negatively or positively influence parents’ treatment of their children.

*“It depends… It could be good to [have] sensitivity to symptoms before they get out of hand. But it could be bad in terms of parental expectation and paranoia and unnecessary reactions.”*

Participants with an interest in a modestly predictive test were more likely to believe that it would have primarily positive or neutral effects on parental treatment of children, compared with participants with no interest (Table 3). An example of a perceived hypothetical risk of testing was that parents might hold their child less responsible for his/her actions.

**Table 3:** Factors related to interest in modestly predictive genetic testing for mood disorder risk in children

	Interested in using a modestly predictive test		Total (%)	Test	*p*-value
	No	Yes			
“Genetic testing for mood disorders in children could affect how parents treat children…” This would be:					
Negative (“bad”)	10	2	12 (24 %)	*χ* ^2^ (2df)	0.010
Neutral/Unsure	5	13	18 (35 %)		
Positive (“good”)	9	12	21 (41 %)		
**Total**	24	27	**51** ^**a**^		
“Genetic testing for mood disorders could lead to discrimination” (job; health or life insurance). This is:					
Unlikely	5	13	18 (37 %)	Exact	0.074
Likely	18	13	31 (63 %)		
**Total**	23	26	**49**		

Shown are the results of Fisher’s exact test (for 2 × 2 tables) or a chi-square test to explore whether responses to each question was associated with an interest in testing children with a modestly predictive genetic test. Results have not been corrected for multiple testing

^a^Note that two participants who did not answer the question about interest in using a modestly predictive test were omitted (one with a negative and one with an unsure response to genetic testing influencing parental treatment of children, see Table 2)

*“Some people will use [the test results] as a crutch,… parents will be more likely to baby them… or make excuses for their behavior.”*

Participants who felt that testing would positively affect parental behavior (40 %) mentioned benefits such as increased sensitivity to children’s moods and actions:

*“I think when you find out there is a depression [predisposition]… you’re a little kinder. You work with [the child] more… You understand some of it’s beyond their control.”*

#### Perceived Risk of Discrimination

Participants were somewhat less likely to be interested in modestly predictive testing if they thought that genetic test results were likely to be used for employment or health insurance discrimination. As shown in Table 3, of the 31 participants who considered employment or health insurance discrimination to be a likely outcome of testing, less than half (42 %) were interested in modestly predictive testing for children, whereas 72 % of the 18 who considered discrimination unlikely were interested in modestly predictive tests (5 participants were unsure about the likelihood of discrimination). Those who were interested in testing were more optimistic that privacy laws and confidentiality would be effective safeguards against discrimination:

*“[Discrimination] is unlikely at this point because results aren’t shared … It’s illegal for them to ask for results.”*

### Preference for Early Testing but not for Early Sharing of Results

Most participants (85 %) said that testing of children for predisposition to mood disorders should occur prior to adolescence, either at birth (21 %; 11/52) or before the age of 12 (65 %; 34/52) (Table 2). The typical explanation for this choice was that knowledge of such a predisposition before adolescence could help parents differentiate between normal teenage behavior and early mood symptoms:

*“Going through the teen years is difficult for any child. And there have always been hormonal issues… Maybe at that point if you knew… a child was more prone to [mood disorder], it may be helpful in sorting out what is just normal adolescence and what maybe has a more serious origin.”*

*“I guess the soonest the kid can spit into a vial…the sooner you know the better.”*

Participants interested in modestly predictive genetic tests for children were also more likely to mention specific medical interventions as potential benefits of testing:

*“We could seek professional help at a younger age, and medication [or] a psychiatrist. Whatever it would take.”*

There was less consensus about whether and when to share results with the child (Table 2). Most participants would either hesitate to share results at all (17 %; 8/48), share them only if symptoms were observed (17 %; 8/48), or delay sharing them until after age 18 (25 %; 12/48). Participants mentioned several potential benefits of sharing test results: increased awareness of possible symptoms, better understanding of their situation if symptoms had started, and feeling less responsible for having problems:

*“I think [test results] would be very helpful. [Children] would know that something was beyond their control…they would know that it’s nothing that they’ve done.”*

Around half (53 %; *N* = 28) of participants endorsed the belief that genetic testing would have a negative effect on a child’s self esteem, mentioning potentially negative consequences such as stigmatization and limiting his/her goals:

*“[Children] can make all kinds of decisions about who they are and what they can be, and limit themselves”*

*“There’s a lot of…stigma associated with mood disorders and… I think it’s gonna negatively affect the way the child views themselves and I don’t think that’s a good thing”.*

### Attitudes Towards Prenatal Testing for Risk of Mood Disorders

Around half of participants (53 %) thought that some people would want to use prenatal genetic testing to make pregnancy termination decisions; 62 % of the entire sample viewed this use as negative, 26 % were unsure, and 11 % viewed it as positive (see Table 2). Negative attitudes were expressed toward prenatal selection against fetuses for any specific characteristics or for mood disorders specifically, and/or toward abortion more broadly. Negative comments addressed the low level of test predictability for illness onset or severity and the treatability of mood disorders.

*“I don’t think there’s going to be a 100 % genetic variable that will assure that if you had a child [he/she] would [be] depressed…I don’t think you shouldn’t have children just because…you might pass that on.”*

Several participants also specifically opposed targeting fetuses with high risk of mood disorders.

“*What kinds of painters and poets would be missing from society if we didn’t have people with mood disorders that see the peaks rather than people that don’t? They are in pain, but it adds to society”*

Those in favor referred to individuals’ right to make their own choice if they had a very severe mood disorder which they did not want to pass on, if the severity of the disorder in the child could be predicted, or if they felt that they could not manage raising a child with such a disorder.

“*Mood disorders can cause a lot of distress…and can seriously impair someone’s life… it would be good in the sense that people would think about the possibilities of the repercussions of having a child that could have that type of mood disorder.”*

## Discussion

Almost all participants in this study expressed interest in using genetic tests with high predictive power for themselves and for their children, but interest was reduced to around 50 % in each case if predictive power was modest (and actual “uptake” would undoubtedly be less, even in similar populations). We have focused here on attitudes toward testing of children. Those who were interested in testing children said that they would do so at birth or before adolescence, and they expressed substantial awareness of potential risks as well as benefits. These findings suggest that there might be significant demand for testing of children if clinically meaningful tests become available. Because these participants had personal and/or family histories of mood disorders and had already joined a personal genomics research project, they might represent more highly motivated “early adopters” of genetic testing of children for mood disorders, and thus their attitudes toward testing might be relevant to the behavior of similar individuals if tests become available.

### Conceptualizing Results Using the Health Belief Model

The discussion incorporates results into the HBM framework (Fig. 1) in order to clarify the relationships among variables that might influence individuals’ future decisions about genetic testing.

#### Perceived Threat of Disease

Most participants perceived a high threat of disease, describing mood disorder as severely affecting themselves and/or family members, and recognizing that their children were at increased risk.

#### Perceived Benefits and Barriers

Those who were interested in modestly predictive tests tended to perceive more benefits and fewer barriers (although this was not statistically significant, see Table 3); they believed that, on balance, benefits of testing (opportunities to obtain effective, early diagnosis and treatment, to provide a more supportive environment for vulnerable children and to facilitate the child’s self-awareness and relief from self-blame) would outweigh the risks (negative changes in parental behaviors and children’s self-esteem, possible violations in confidentiality with potential for resulting discrimination). Although a greater proportion of participants expressed interest in highly predictive tests, approximately half still favored using a test with modest power. Consistent with previous research, qualitative responses suggest that participants who did not favor use of modestly predictive tests were less likely to endorse genetic factors in the etiology of mood disorders, had less interest in or rejected medical treatment of mood disorders, had greater concern about loss of confidentiality and resulting discrimination, and had greater worries about negative effects on parental behavior and children’s self-image (Meiser et al. 2005). Similar expected benefits and risks of genetic testing for psychiatric disorders have been reported in studies of researchers and psychiatrists (Erickson and Cho 2011; Hoop et al. 2010; Miklowitz and Chang 2008); and also for non-psychiatric later-onset diseases (Mand et al. 2012). Participants who supported testing before adolescence identified the ability to identify and thus provide support and treatment for at-risk children before that difficult period as a benefit. Although participants acknowledged negative aspects of early testing, such as potential negative impact on children’s self-image, these were viewed as manageable by not disclosing results to the child unless symptoms emerged. It is unclear how effective this strategy would be, however, since keeping test results from a child could be difficult for a parent.

#### Likelihood of Adopting the Health Action (Testing Children)

These results suggest that some parents will consider using a modestly predictive genetic test for mood disorder predisposition if they believe that mood disorders are severe diseases for which they can transmit an increased genetic susceptibility, and if they judge the expected benefits to an individual child to outweigh the perceived risks. The high level of interest in such tests in this sample (51 %) was probably (and intentionally) influenced by our recruitment of members of a personalized medicine research program. While attitudes held by key individuals (spouses, family members, physicians) are sometimes found to impact decision-making and intent toward action in other contexts, most of our participants stated that they felt free to act on their intentions regardless of others’ opinions. Note that our interview was structured such that questions about potential risks and ethical, legal and social implications were asked before questions about interest in testing for self or children. This might have led to more realistic, well-considered responses regarding interest in testing. From these findings we hypothesize that among people with a strong interest in genetics and personalized medicine, there will be “early adopters” of any future genetic tests for mood disorders in children, and that this population might be somewhat resistant to social pressures against testing.

### Comparison with Previous Studies

In this sample of individuals who were already favorable toward genetic testing, a good predictor of interest was the predictive power of the test. In contrast to tests for Mendelian dominant or recessive disorders, genetic tests for mood disorders might yield only weak estimates of predisposition because of the complex interplay of genetic and environmental factors (Braff and Freedman 2008; Wright and Kroese 2010); yet, half of our participants were interested in a hypothetical test with a modest (20 %) predictive power.

Our results support and extend those of six previous studies around attitudes towards genetic testing for psychiatric illness. One study (Smith et al. 1996) reported that 89 % of 48 members of BPD support groups would want definitive genetic testing for their children, assuming availability of preventive treatment. A second study (Trippitelli et al. 1998) reported that 78 % of 40 BPD patients (from a genetic study of multiply-affected families, a support group and clinical patients) and “a majority” of their spouses would want definitive testing for children. Jones et al. (Jones et al. 2002) reported that 78 % of 147 BPD patients (most from a genetic study of multiply-affected families) favored testing; the question did not mention predictive power and thus implied a definitive test. “Approximately half” of 22 members of multiply-affected families from a genetic study of BPD reported that they would test adolescents, citing early treatment as a benefit and change in parental behavior as a risk (Meiser et al. 2005). In a subsequent survey of 95 BPD and 105 unaffected members of multiply-affected families, 80 % were definitely or probably interested in testing their children using a definitive test, with participants expressing less interest in a non-definitive test for themselves (Meiser et al. 2008). Similar to our study, the preferred age of testing was at birth (30 %), early childhood (33 %) or between ages 10 to 17 (27 %), vs. only 9 % for over 18. Finally, in a community-based sample, 72 % of 228 participants with MDD and 55 % with BPD favored definitive genetic testing for their children, dropping to 26 % and 31 % respectively if no effective treatment or prevention was available (Laegsgaard et al. 2009).

Thus, previous studies asked only about interest in *definitive* testing for children, and all but one were limited to BPD patients. Benefits and risks of child testing were queried briefly or not at all. When similar issues were studied, results were generally comparable to ours. Of note, four of the six previous studies were conducted five or more years ago. Given the rapid developments in genetics and in public awareness of genetics over the past decade, interest in and attitudes towards genetic testing are likely to be continually evolving.

### Limitations

Our sample was small, mostly female and not representative of the general population; we cannot comment on how these results would compare with the attitudes of individuals from other ethnic groups, with less education, with no personal or family history experience with MDD or BPD, or with less knowledge about and interest in genetics. Studies of attitudes toward hypothetical predictive genetic tests have historically over-estimated the eventual use of real tests (Lerman et al. 2002). We attempted to mitigate this effect by studying individuals who are already participating in a personalized medicine program, but it is not known to what extent their responses are more likely to reflect actual future behavior.

### Conclusions and Implications for Clinical Intervention, Research and Policy

Our results, using mood disorders as an example, suggest that there will be substantial interest in testing children for a modestly increased genetic susceptibility to genetically complex disorders that fall between the classical dichotomy of “actionable” disorders (for which specific treatments or preventive measures are available for children) vs. late-onset disorders with no useful early intervention (American Society of Human Genetics 1995; Borry et al. 2006; Ross et al. 2013; National Society of Genetic Counselors 2012). Mood disorders in particular fall into this gray area. Although there is no definitive early treatment for mood disorders, controlled trials have demonstrated reduction of symptom severity or of probability of clinical onset during follow-up in children who were considered to be at high risk because of symptoms and behavior and who received school- or family-based psychotherapies (e.g., Arnarson and Craighead 2011; Stice et al. 2010; Garber et al. 2009; Miklowitz and Chang 2008). Some parents will be interested in having children tested early in life for their predisposition to a condition that has been diagnosed in one or more close relatives, even if predictive power is modest, age of onset is highly variable and options for prevention or treatment remain uncertain. The actual number of parents wishing to test their children might initially be small, but this will still present health professionals (particularly genetic counselors) and society at large with a set of new and challenging issues (Tercyak et al. 2011). We would expect interest in testing to increase as the general public becomes more knowledgeable about genetics and more aware of privacy protections for genetic information.

A critical issue raised by our results is whether and how children would be involved in the decision to be tested and in the discussion of results. Our participants generally wanted to test children before adolescence, but not share results with the child until adulthood or until symptoms developed—i.e., parents alone would make decisions about testing. Interestingly, in a study of presymptomatic testing for alpha-1 antitrypsin deficiency, parents were generally resistant to involving children in consenting to testing; but adults who had actually been tested as children for this disease were significantly more likely to believe that the child should be involved in the consent process (Coors et al. 2011). Bioethicists disagree about whether the emphasis here should be on the right of parents to make decisions in the child’s best interest, the child’s own right to autonomy, or a family-based process (Fenwick 2010). This ambiguity regarding how and when children should be involved in the decision-making process for genetic testing will become particularly critical for late-onset and potentially stigmatizing disorders such as psychiatric conditions. For example, how will a late adolescent or young adult react to being told by parents that they had altered their treatment of the child after a test at age 10 had suggested an elevated risk of a mood disorder, although they had not disclosed those results to the child? We share the view—expressed by some of our participants—that, on the one hand, being told at an early age that one is genetically predisposed to mood disorder could be a source of stress and self-doubt, but that it could also contribute to constructive efforts by some children and their parents to educate themselves, to address sources of stress, and to seek early treatment if symptoms develop. As genetic testing becomes available to predict adult onset of conditions, the issues of how children should be involved in genetic testing decision-making as well as the potential developmental risks and benefits of early testing for children will have to be confronted and special protocols for genetic counselors working with this age-group will be needed.

Predictive power is a key factor in making decisions about testing, and even more so for parental decisions about testing children. Our study showed that participants were able to differentiate between modest and highly predictive genetic tests, but many were interested in both. Many geneticists who study complex disorders are skeptical about the prospects for predispositional testing for complex diseases, and point out that in most cases family history alone provides more meaningful information about risk than any genetic information (Do et al. 2012; Paynter et al. 2009). In fact, at this point in time, family history still remains the gold standard for predicting risk of psychiatric disorders. However, individuals may respond differently to individual DNA tests than to family history information. For example, women who learned that they had a 29 % lifetime risk of Alzheimer’s disease based on ApoE4-negative genotype reported a much more positive response to this information as well as reduced perception of risk and reduced anxiety, compared with women who learned that their risk was 29 % based only on family history and gender (LaRusse et al. 2005). Given this differential perception and the interest observed in our population and others, it is likely that regardless of predictive power, genetic testing for complex diseases will be used by some, if not broadly, when available.

Because of the large size of the potential market for genetic testing for common, complex disorders, it will be tempting for companies to market tests by exaggerating their predictive power, but consumers and clinicians need accurate information. There are several scenarios involving testing that could arise in the near future. One is that an essentially useless test (e.g., less predictive than family history information alone) could be marketed, and that some parents might succeed in having children tested. Here, the response of medical professionals would be straightforward: discourage use of such a test, help families to understand its lack of validity, and initiate discussion about the issues that led to the interest in testing. A second scenario is that a test with some meaningful (if modest) degree of predictive power would become available, either by prescription or direct-to-the-consumer, and would be used to test some children. In this scenario, medical professionals should help patients understand risk. Genetic concepts and especially numerical estimates of relative and absolute risk or probability are quite difficult to communicate (Austin et al. 2012; Lea et al. 2011; Austin 2010; Marteau 1999). We found that our study participants had difficulty understanding the concept of risk prediction even when worded in a fairly simple manner. Tests may be perceived by consumers as having *personal utility*, even if predictive power and thus clinical utility are relatively low (Biesecker and Peay 2013). More research is needed to establish how best to communicate the concept of genetic risk or probability effectively. In the case of mood disorders, many people would like to know *which* of their children are at greatest and lowest risk (rather than simply that the risk to all of their children is elevated on average), and they may feel that a little information is better than none. Both of these scenarios require significant education among clinicians regarding the value, validity, and predictive power of each test, and this may be particularly true for psychiatric disorders. In both of these cases, there is a need for education for consumers and clinicians, for an ethical debate on the value, benefits and risks of these interventions, and regulations regarding the availability of these tests.

Finally, most health professionals would agree that it is desirable to test strategies for preventing illness in at-risk children, including psychosocial interventions. In this context, if there were a valid genetic test to identify groups of offspring who had higher or lower degrees of genetic risk, it could make sense to stratify the child research participants according to degree of risk and to determine whether risk estimates were (positive or negative) predictors of the effectiveness of the intervention . Such a study would be the research equivalent of what some parents might wish to do on their own: identify children at increased risk, and pay particular attention to helping them to build self-esteem and cope with stress. Based on our research, we would recommend that if a genetic testing protocol includes disclosure of results of genetic testing of (and possibly to) children, the research should assess both short- and long-term outcomes in parents and children, and should help clinicians to develop an evidence-based approach to future practice.

In conclusion, our results suggest that there are several clinical, ethical and policy issues that need further consideration to help health professionals, policymakers, families and children themselves respond to the challenges that will be posed by future availability of tests for genetic risks of mood disorders. It is often difficult for family members to decide whether and how to communicate with each other about genetic risk, regardless of the nature of the disease (Metcalfe et al. 2011). We currently know little about how to help families to make these decisions in ways that maximize good outcomes.

## Electronic supplementary material

Below is the link to the electronic supplementary material.

ESM 1 (DOC 87 kb)
