# Supplementary material for: Genetic Testing of Children for Predisposition to Mood Disorders: Anticipating the Clinical Issues
Source: J Genet Couns. 2014 Aug 1;23(4):566–77. doi: 10.1007/s10897-014-9710-y (PMC4090807; doi:10.1007/s10897-014-9710-y)
Supplement: Supplementary file 1 — (DOC 87 kb) [file 10897_2014_9710_MOESM1_ESM.doc]

The following interview schedule was used to interview participants in the study that has been initially reported in Erickson et al., Genetic Testing of Children for Risk of Mood Disorders: Anticipating the Clinical Issues, Journal of Genetic Counseling, in press, 2014. The questions deal with issues related to testing for onself as well as for children. Questions that are directly relevant to the results reported in this paper are highlighted in yellow. Please contact Dr. Douglas Levinson, [dflev@stanford.edu](mailto:dflev@stanford.edu), with any questions.

**Interview Schedule on Genetic Testing for Mood Disorders**

Lili Kuzmich, Jessica Erickson, Kelly Ormond, Mildred Cho and Douglas Levinson

Stanford University Center for Integrative Research on Genetics and Ethics

Participant ID:__________________ Date:____________ Interviewer:________________

**INTRODUCTION:**

I) Thanks for agreeing to participate.

II) Background:

This study is being conducted by researchers from Stanford University and the Coriell Institute. We are interviewing people who have a personal or family history of mood disorders to learn more about attitudes and interest in genetic testing for mood disorders This interview should take between 60-120 minutes.

III) Informed consent:

You have already read the online consent form and agreed to participate in this interview study. Do you have any additional questions? As explained in the consent, we will be recording and transcribing this interview, but we will remove your name and any additional information from the interview answer forms that might identify you. You can stop the interview at any time, and you can refuse to answer any questions. You can also ask to have your interview removed from the study if you like. Do you still want to participate in the study? Do I have your permission to start recording?

□ Participant confirms agreement to interview and recording.

□ Does not agree. Comment:_________________________________________________

_________________________________ _________

Interviewer signature Date

**Demographics:**

1. How old are you?
2. What is your ethnicity?
3. Are you single, with a partner, or married?
4. Do you have children? How old are they?
5. What is the highest level of education you have completed?
6. Are you employed?
7. What is your career/field of work?

8. In this interview, I am going to refer to certain mental health problems as “mood disorders.” Have you heard this term? What kinds of mental health problems do you understand this term to include? [Assess the participant’s level of information, provide clarification if necessary that “mood disorders include “depression in the sense of major depression or “clinical” depression, and bipolar disorder which is sometimes known as manic-depression”, and continue to clarify briefly if the participant seems unfamiliar with these terms. Rate the participant’s ability to understand the concept with regard to the remaining questions in this interview:
 -Almost no understanding;
 -Limited: questionable ability to understand the interview;
 -Fair: has some knowledge but might misunderstand some questions;

-Good: has adequate knowledge to understand the questions;

-Excellent: very well-informed, high level of knowledge

9. I am also going to use the term “genes.” What is your understanding of what genes are?

[Assess participant’s level of information. Provide clarification if needed that “genes are DNA -- the chemical in cells in the body which you inherit from parents and which direct how your body is made; genes are why people might resemble parents or other family members more closely in terms of height, appearance, temperament, etc. Rate the participant’s ability to understand the concept with regard to the remaining questions in this interview:
 -Almost no understanding;
 -Limited: questionable ability to understand the interview;
 -Fair: has some knowledge but might misunderstand some questions;

-Good: has adequate knowledge to understand the questions;

-Excellent: very well-informed, high level of knowledge

**Personal and family history:**

Have you ever been diagnosed with a mood disorder?

10. What type of mood disorder do you have?

a. Have you been diagnosed with a psychiatric disorder? What diagnosis did you receive?

__Major depression __Bipolar disorder-I __Bipolar disorder-II __Other depression

Briefly describe:

b. Who made the diagnosis?

__Psychiatrist __Therapist/psychologist __Non-psychiatrist MD __Other

c. How old were you when you first had a serious problem with depression or mania?

Age at onset____

d. How many depressive or manic episodes have you had in your life?:

Number of episodes____ or chronic____

Can you tell me a little about what your periods of [depression] [mania] [depression and mania] have been like? At their worst, how have they affected your life and on your ability to function?

e. Severity of the worst episodes:

___Disabling (cannot function in major roles such as job, family life)

___Substantial decline in functioning in major roles

___Could function in major roles but less well than usual

___No substantial impact on major roles

f. To what extent do things get better for you between depressive or manic periods?

___Full remission

___Mild mood or related symptoms in between, but mostly remitted

___Improved, but substantial mood-related symptoms remain

___Symptoms are essentially chronic

g. What treatment have you had?

___Medication ___Psychotherapy/counseling ___ECT ___Other

Describe:

1. Has anyone in your family been affected by a mood disorder or suicide? Who? [Define anyone as grandparents, parents, aunts/uncles, cousins, children, and spouses/partners who are parents of any of the subject’s children.]

For each individual mentioned, interviewer will then probe for the following information, rate it in a grid format, and record notes about the subject’s comments:

a. Basis of report (observing symptoms; hearing about diagnosis; hearing about treatment)

b. Reliability of the report (interviewer’s global impression)

c. Type of mood disorder

d. Duration

e. If treated, perceived effectiveness of treatment

f. Severity – how severe would you say your family member’s mood disorder was? What is this based on? Did it impact their daily life? Relationships? Ability to work?

12. Global severity of family history: _____ None _____ Mild _____ Moderate _____ Severe

1. a. **If FH+:** did the mood disorders of other(s) in your family have an effect on you? What was the effect?

b. Global severity of FH+: _____ None _____ Mild _____ Moderate _____ Severe

**Belief system about mood disorder:**

1. a. People attribute mood disorders to a variety of factors. These factors include: someone’s genes; or someone’s experiences such as: stress, childhood events, or drug abuse; or by a combination of both genes and experiences. In your opinion, what are mood disorders in most people caused by?

PROMPTS:

- almost entirely by genes?
- more by genes but with some effects of experiences?
- by both genes and experiences - around 50-50?
- more by experiences but with some effects of genes?
- almost entirely by experiences?

1. Are there any other factors which you think determine whether someone will have a mood disorder? [describe:]
2. a. From what you have seen and heard, around what percentage of people in the whole population, on average, will have a mood disorder at some point in their lives?

b. -And around what percentage will develop a severe enough mood disorder that makes family and work life much more difficult?

Refer to question #4 in demographics section to determine whether the subject has children and then use the appropriate subsection below:

**If has children:**

1. (If under 50): Are you considering having any more children and if so, do you have an idea of when?
2. Do you have any concerns that any of your children might be showing signs of having or developing a mood disorder? **If yes,** What makes you think that? What types of symptoms is he/she having?

Describe:

1. a. What do you think your children’s probability of developing a mood disorder is?

Prompts:

- Around the same as the average person in the population?
- More than the average person?
- Just slightly more (10-50% greater probability)?
- Around twice as likely?
- More than twice as likely? [ask how much]
- Less than the average person?

b. What makes you think that their risk is [restate risk they provided]?

1. a. If one of your children developed a mood disorder, what do you think the severity would be?

__Mild __ Moderate __Severe

b. -What type of effect do you think it would have on his or her life? [prompts: What makes you think that it would be mild/severe/very severe if it happened?]

**If no children but considering:**

1. Are you considering having children?
2. a. For someone like yourself [refer to presence/absence of mood disorder and of family history], do you think that if you had children, their probability of developing a mood disorder at some point in their lives is:

PROMPTS:

- Around the same as the average person in the population?
- More than the average person?
- Just slightly more (10-50% greater probability)?
- Around twice as likely?
- More than twice as likely? [ask how much]
- Less than the average person?

b. What makes you think that their risk is [restate risk they provided]?

1. If one of your children developed a mood disorder, do you think it would be mild and have little effect on their lives overall, or severe with a major effect on their lives, or somewhere in between? [What makes you think that it would be mild/severe/very severe if it happened?]

Mild; Moderate; Severe

**If not considering:**

1. Has the mood disorder in your family had any influence on your decision to not have children?

If yes, in what way?

1. a. For someone like yourself [refer to presence/absence of mood disorder and of family history], do you think that if you had children, their probability of developing a mood disorder at some point in their lives is:

- Around the same as the average person in the population?
- More than the average person?
- Just slightly more (10-50% greater probability)?
- Around twice as likely?
- More than twice as likely? [ask how much]
- Less than the average person?

b. What makes you think that their risk is [restate risk they provided]?

**Stage of awareness of genetics and genetic testing:**

[Note -- assessment of risks and attribution to genes have been asked above, because they should come before asking about children’s risks.]

“I am now going to ask you some questions that refer to the idea of “genetic testing.” As you may know from your participation in the Coriell Personalized Medicine program (CPMC), genetic testing can be done to look for the risk of a complex disease, a disease that is caused by a combination of multiple genes and the environment, as is done in the CPMC, or genetic testing can be done to look for diseases that are caused by a single gene like cystic fibrosis or muscular dystrophy (the CPMC does not test for single gene disorders).

Right now, there are no genetic tests that are known to predict whether someone will develop a mood disorder. But researchers have identified some genetic differences between people with and without bipolar disorder that seem to predict whether someone might have a small increase in risk, like, being 10 or 15% more likely to develop bipolar disorder than the average person.

1. Have you heard about this kind of genetic predictor of any type of mood disorder?

Yes No [describe:]

1. There are some companies that sell genetic Tests that include information about some of these genetic predictors of mood disorders.

a. -Have you heard about these tests? Yes No [describe:]

**If yes,**

b. -Have you ever had this kind of test? [yes; no; describe]

c. -**If yes**: Was your decision to have this test at all influenced by an interest in learning more about genetic risks of mood disorders? [yes; no; probe and record comments]

c. -Have you ever considered having this kind of test? [Yes; no; describe]

If yes, Why?

d. -Is your interest in possibly having such a test at all influenced by an interest in learning more about genetic risks of mood disorders? [yes; no; probe and record comments]

**If interviewee has not heard of DTC testing**:

e. -Would you be interested in a test like this? Why or why not?

27. a. -I know that you have participated in the Coriell Personalized Medicine program. Have you participated in any other programs where you gave blood or saliva for genetic studies?

No Yes

b. -Was your interest in [this program] [these programs] at all influenced by an interest in learning more about genetic risks of mood disorders?

No Yes [probe and record comments]

We’ve been told that a number of CPMC participants have particular disease interests. Although Coriell is interested in learning more about what types of testing participants are interested in, we have been asked to mention that Coriell has no plans to include genetic testing for mood disorders at this time.

**Attitudes about genetic testing for mood disorder**

1. What are your thoughts and opinions about the idea of using genetic tests to predict who will develop mood disorders? Do you think that it is generally a good or a bad thing to try to develop such tests? What makes you think so? [Record comments, and rate on scale]

1=very negative

2=somewhat negative

3=neutral, not sure

4=somewhat positive

5=very positive

1. OK. Now, I would like to ask your opinion about predictions that some people have made about how genetic tests for mood disorders might be used in the future, and what their positive and negative effects might be. It is not yet known exactly how well these tests will be able to predict who will develop mood disorders, so there are no right or wrong answers here, I am just interested in your opinions based on your personal knowledge and experience. I am going to read a list of predictions that different people have made. For each one, I will ask you how likely you think it is, whether you think it would be a positive or negative thing, and whether you think it might influence whether you or people like you might have a genetic test for mood disorders or request it for a child in the future.

Even though I will be asking you specific prompts after each scenario, we are mostly interested in hearing your thoughts and opinions so please feel free to elaborate after each prompt.

1. **Genetic tests for mood disorders might permit earlier diagnosis.**

- How likely do you think this is? [very unlikely, unlikely, neutral/unsure, likely, very likely]
- If it turned out to be true, would this be a good or a bad thing? (very good, somewhat good, neutral/unsure, bad, very bad]
- If it turned out to be true, how do you think it would influence whether people like you would request such a test For yourself? For a child?

1. **Genetic tests could make it more possible to prevent a mood disorder before it starts. For example, in a young person who has not had any symptoms, genetic test results might lead to treatment to prevent them from ever developing a mood disorder.**

- How likely do you think this is? [very unlikely, unlikely, neutral/unsure, likely, very likely]
- If it turned out to be true, would this be a good or a bad thing? (very good, somewhat good, neutral/unsure, bad, very bad]
- If it turned out to be true, how do you think it would influence whether people like you would request such a test For yourself? For a child?

1. **Genetic tests for mood disorder could permit earlier treatment in someone who has started to have symptoms. For example in a person who has already had some symptoms of a mood disorder but not a definite dx, genetic test results could help clarify dx and begin earlier treatment.**

- How likely do you think this is? [very unlikely, unlikely, neutral/unsure, likely, very likely]
- If it turned out to be true, would this be a good or a bad thing? (very good, somewhat good, neutral/unsure, bad, very bad]
- If it turned out to be true, how do you think it would influence whether people like you would request such a test For yourself? For a child?

1. **Genetic tests could help doctors to pick the best treatment for an individual depending on their genetic differences.**

- How likely do you think this is? [very unlikely, unlikely, neutral/unsure, likely, very likely]
- If it turned out to be true, would this be a good or a bad thing? (very good, somewhat good, neutral/unsure, bad, very bad]
- If it turned out to be true, how do you think it would influence whether people like you would request such a test For yourself? For a child?

1. **Genetic testing for mood disorders could help a person to plan for the future.**

- How likely do you think this is? [very unlikely, unlikely, neutral/unsure, likely, very likely]
- If it turned out to be true, would this be a good or a bad thing? (very good, somewhat good, neutral/unsure, bad, very bad]
- If it turned out to be true, how do you think it would influence whether people like you would request such a test For yourself? For a child?

1. **Genetic testing for mood disorders might influence some people in making decisions about whether or not to have children.**

- How likely do you think this is? [very unlikely, unlikely, neutral/unsure, likely, very likely]
- If it turned out to be true, would this be a good or a bad thing? (very good, somewhat good, neutral/unsure, bad, very bad]
- If it turned out to be true, how do you think it would influence whether people like you would request such a test For yourself?

1. **Genetic testing for mood disorders in children could affect how parents treat children depending on their test results?**

- How likely do you think this is? [very unlikely, unlikely, neutral/unsure, likely, very likely]
- If it turned out to be true, would this be a good or a bad thing? (very good, somewhat good, neutral/unsure, bad, very bad]
- If it turned out to be true, how do you think it would influence whether people like you would request such a test For a child?

1. **The results of a genetic test for mood disorders in children could shape a child’s view of him or herself? [and probe what effects the participant would predict and would view positively or negatively]**

- How likely do you think this is? [very unlikely, unlikely, neutral/unsure, likely, very likely]
- If it turned out to be true, would this be a good or a bad thing? (very good, somewhat good, neutral/unsure, bad, very bad]
- If it turned out to be true, how do you think it would influence whether people like you would request such a test For a child?

1. **If prenatal genetic testing for mood disorder became available, it could affect someone’s decision to terminate a pregnancy.**

- How likely do you think this is? [very unlikely, unlikely, neutral/unsure, likely, very likely]
- If it turned out to be true, would this be a good or a bad thing? (very good, somewhat good, neutral/unsure, bad, very bad]
- If it turned out to be true, how do you think it would influence whether people like you would request such a test For yourself?

***Don’t need to ask if “good thing or bad thing” for the following predictions:***

1. **Genetic testing for mood disorders could increase stigma against those with tests predicting higher risk. By stigma I mean being judged negatively by other people just because of that one fact.** *If interview subject raises privacy issues, then acknowledge that privacy acts do indeed exist, but that some people still have concerns that information could be wrongfully accessed, and we are trying to learn more about people’s concerns if any.*

- How likely do you think this is? [very unlikely, unlikely, neutral/unsure, likely, very likely]
- If it turned out to be true, how do you think it would influence whether people like you would request such a test For yourself? For a child?

1. **Genetic testing could increase someone’s worry and anxiety if they were found to be at higher genetic risk for developing a mood disorder.**

- How likely do you think this is? [very unlikely, unlikely, neutral/unsure, likely, very likely]
- If it turned out to be true, how do you think it would influence whether people like you would request such a test For yourself? For a child?

1. **Genetic testing for mood disorders could lead to discrimination in getting hired for a job or in obtaining health or life insurance.**

- How likely do you think this is? [very unlikely, unlikely, neutral/unsure, likely, very likely]
- If it turned out to be true, how do you think it would influence whether people like you would request such a test For yourself? For a child?

1. **Someone who found out that they had a higher risk of a mood disorder might be more likely to feel stressed, depressed, or vulnerable.**

- How likely do you think this is? [very unlikely, unlikely, neutral/unsure, likely, very likely]
- If it turned out to be true, how do you think it would influence whether people like you would request such a test For yourself? For a child?

1. **Someone who found out that they had a high risk of a mood disorder might have an increased risk of suicide.**

- How likely do you think this is? [very unlikely, unlikely, neutral/unsure, likely, very likely]
- If it turned out to be true, how do you think it would influence whether people like you would request such a test For yourself? For a child?

1. Now I am going to ask you some questions about what you think might happen in the future if you had the possibility of taking a genetic test for mood disorders. *(These are all hypothetical situations but we are still interested in hearing your opinion to these situations)*

-How confident do you feel that you would follow through and actually get tested?

- Definitely would
- more likely to do it
- 50-50
- more likely not to do it
- definitely would not do it
- not interested

31. What barriers would you foresee that might keep you from following through on a decision to get such a test? [probe and record comments]

[Will provide list of possible barriers including financial, others’ reactions, stigma, etc.]

- Financial (cost; lack of insurance coverage; test not covered by insurance)
- Concern about discrimination based on results (insurance, employment)
- Might put it off because of worrying about the possible result
- Worry about others’ reactions [who? Spouse/partner, other family members, etc.]
- Concern about possible stigma [being looked at negatively because of the result]
- Concern about children’s reaction

**Subjective Norms:**

1. If genetic testing for mood disorders became available in the future, are there individuals in your life, such as family, friends or doctors, whose opinions you think might influence whether you personally would get tested?

-No one else would have much influence

-Spouse/partner; Children; Friends; Medical doctor; Psychiatrist; Therapist; Clergyman; Other (specify)

[For each one, probe and record comments; and rate the participant’s prediction of

a) Whether the reaction to testing would be positive, neutral or negative; and

b) Whether that person’s reaction would make the participant more or less likely to be tested or no effect.

33. If genetic testing for mood disorders became available in the future, are there individuals in your life, such as family, friends or doctors, whose opinions you think might influence whether you would get a child tested?

-No one else would have much influence

-Spouse/partner; Children; Friends; Medical doctor; Psychiatrist; Therapist; Clergyman; Other (specify)

[For each one, probe and record comments; and rate the participant’s prediction of

a) Whether the reaction to testing would be positive, neutral or negative; and

b) Whether that person’s reaction would make the participant more or less likely to be tested or no effect.

**Evaluation of genetic testing based on predictive power:**

It is not yet clear how well any future genetic tests might be able to predict someone’s chances of developing a mood disorder. Many people think of a genetic test as being either 100% positive or 100% negative. But a test might only determine who might be at an increased risk for a mood disorder without being able to predict exactly who would develop one.

Lets imagine that some future genetic testing program could either predict with a high degree of certainty (lets say 90%) that someone would go on to develop a mood disorder or could only predict with a moderate degree of certainty (let say 20%) that someone would go on to develop a mood disorder.

1. a. How interested do you think you would be in having a genetic test for a mood disorder, if it could predict with a high degree of certainty that people with a positive result would develop a mood disorder?

● Would definitely be interested

● Might be interested

● Would probably not be interested

● Definitely would not be interested

- b. How do you think it might help someone to know that they were very likely to develop a mood disorder? [probe and record]

c. What problems might it cause if someone knew they had a high risk of developing a mood disorder? [probe and record]

34 d. How interested do you think you would be in having a genetic test for a mood disorder, if it could predict with only a modest degree of certainty that people with a positive result would develop a mood disorder?

● Would definitely be interested

● Might be interested

● Would probably not be interested

● Definitely would not be interested

- e. How do you think it might help someone to know that they had a moderately increased risk of developing a mood disorder? [probe and record]
- f. What problems might it cause if someone knew they had a moderately increased risk? [probe and comment]

1. *If the participant has, or is at all interested in having, children -- if interest is questionable, ask participant if he/she would like to discuss this topic.*

a. How interested would you be in having a child tested if the test could predict with a high degree of certainty that people with a positive result would develop a mood disorder?

● Would definitely be interested

● Might be interested

● Would probably not be interested

● Definitely would not be interested

- b. How do you think it might be helpful for you as a parent to know that a child had a high risk? [probe and record -- issues that might come up, and should be probed, include being on the lookout for symptoms, possible early treatment with medication and/or counseling, and whether or not a parent would consider treating a child differently]
- c. How do you think it might be problematic for you as a parent to know that a child had a high risk? What reservations might you have? [probe and record]

d.. How interested would you be in having a child tested if it could predict only with a modest degree of certainty that people with a positive result would develop a mood disorder?

[rate interested, no interested, unsure]

● Would definitely be interested

● Might be interested

● Would probably not be interested

● Definitely would not be interested

e. How do you think it might be helpful for you as a parent to know that a child had a moderately increased risk? [probe and record -- see issues under item 30]

f. How do you think it might be problematic for you as a parent to know that a child had a (moderate or high) risk? What reservations might you have? [probe and record]

36. What do you think would be the best age to have a child tested? Why?

37. Let’s assume that the child was tested at age [the age given in response to 30c - or indicate if the participant prefers to discuss a different age]:

a. Do you think that you would consider discussing the results with the child at some point if the test indicated an increased risk, and if so do you think that this would be at the age of testing, or at a later age?

Would be most likely to:

● never discuss with the child

● discuss at the age of testing

● discuss later, at around ______ years of age

[Probe and record details]

b. How do you think that it might be helpful to the child to learn about the results? (*note: participant might have some thoughts about possible benefits, even if participant would be unlikely to actually discuss)*

c. Are there ways in which it might be harmful or distressing to the child to learn about the results? What reservations would you have about doing this?

d. Do you think that the degree of certainty about the child’s risk might be a factor in your decision -- for example, if a test indicated a very high risk such as 90%, vs. a test that indicated only a moderate certainty about the child’s risk such as 20 or 30 or 40%?

1. a. Compared to other disorders such as heart disease, diabetes or cancer, how interested would you be in having genetic testing for a mood disorder? Why would you be more interested in one vs. another?

__Less interested in tests for mood disorders than in tests for medical diseases

__About the same

__More interested in tests for mood disorders

1. Compared to other disorders such as heart disease, diabetes or cancer how interested would you be in having your child tested for a mood disorder? Why would you be more interested in one vs. another?

__Less interested in tests for mood disorders than in tests for medical diseases

__About the same

__More interested in tests for mood disorders
